# microRNA-210-3p depletion by CRISPR/Cas9 promoted tumorigenesis through revival of TWIST1 in renal cell carcinoma

**DOI:** 10.18632/oncotarget.14930

**Published:** 2017-02-01

**Authors:** Hirofumi Yoshino, Masaya Yonemori, Kazutaka Miyamoto, Syuichi Tatarano, Satoshi Kofuji, Nijiro Nohata, Masayuki Nakagawa, Hideki Enokida

**Affiliations:** ^1^ Department of Urology, Graduate School of Medical and Dental Sciences, Kagoshima University, Kagoshima 890-8520, Japan; ^2^ Department of Internal Medicine, Vontz Center, University of Cincinnati, College of Medicine, Cincinnati, OH 45267-0508, USA; ^3^ Moores UCSD Cancer Center, La Jolla, CA 92093-0803, USA

**Keywords:** microRNA, *miR-210-3p*, renal cell carcinoma, CRISPR/Cas9, TWIST1

## Abstract

Previous studies showed that five miRNAs (*miR-885-5p*, *miR-1274*, *miR-210-3p*, *miR-224* and *miR-1290*) were upregulated the most in clear cell renal cell carcinoma (ccRCC). Our focus was to understand from a clinical standpoint the functional consequences of upregulating *miR-210-3p*. Towards this, we utilized the CRISPR/Cas9 gene editing system to deplete *miR-210-3p* in RCC cell lines (786-o, A498 and Caki2) and characterized the outcomes. We observed that *miR-210-3p* depletion dramatically increased tumorigenesis, including altering the morphology *of* A498 and Caki2 cells in a manner characteristic of epithelial-mesenchymal transition (EMT). These results were corroborated by *in vivo* xenograft studies, which showed enhanced growth of tumors from *miR-210-3p*-depleted A498 cells. We identified Twist-related protein 1 (*TWIST1*) as a key target of *miR-210-3p*. Analysis of the ccRCC patient data in The Cancer Genome Atlas database showed a negative correlation between *miR-210-3p* and *TWIST1* expression. High *TWIST1* and low *miR-210-3p* expression associated with poorer overall and disease-free survival as compared to low *TWIST1* and high *miR-210-3p* expression. These findings suggest that renal cell carcinoma progression is promoted by *TWIST1* suppression mediated by *miR-210-3p*.

## INTRODUCTION

Nearly 80% of renal cell carcinoma (RCC), a malignant cancer of the tubular cells of the kidney, is clear cell RCC (ccRCC) [1]. The advanced-stage RCC has a poor 5 year survival rate (5–10%) due to recurrence or distant metastasis and nearly 30% of RCCs are diagnosed with metastasized cancers [2]. The current treatments for RCC include molecularly targeted therapeutics such as multi-targeted receptor tyrosine kinase (RTK) or mTOR inhibitors that are widely used for patients with metastatic or recurrent RCC. However, these types of therapeutics delay cancer progression rather than curing it [3, 4]. Therefore, there is greater impetus to elucidate the molecular mechanisms underlying RCC development and progression so that novel therapeutic targets can be identified.

MicroRNAs (miRNAs) are endogenous small non-coding RNAs (ncRNAs) that are 19–22 bases in length and regulate gene expression by repressing translation or cleaving RNA transcripts in a sequence-specific manner [5]. Nearly 2585 mature human miRNAs are registered in miRBase (release 21, June 2014;http://www.mirbase.org/). More than 60% of the protein-coding genes in the human genome are predicted to be regulated by miRNAs [6]. Many miRNAs are found to be aberrantly expressed in a number of human cancers and the role of few miRNAs in regulating oncogenesis and metastasis has been studied [7, 8]. Therefore, investigating the cause and function of aberrantly expressed miRNAs is important to elucidate the clinical relevance of miRNA-mediated oncogenic pathways in tumorigenesis.

We previously reported several miRNAs that were associated with cancer cell proliferation, migration and invasion [9, 10]. Our primary focus had been to study the downregulated miRNAs that functioned as tumor suppressors. Although many studies have successfully inhibited miRNA expression by using miRNA inhibitors, morpholinos, or sponges, the knock down efficiency has not been fully satisfactory with off-target effects [11–13]. Neither has the cause and effect of upregulated miRNAs been fully understood so that they could be used as cancer markers [14].

Clustered regulatory interspaced short palindromic repeats (CRISPR) were first discovered in 1987 [15]. The basic CRISPR components include the CRISPR RNA (crRNA) and the endonuclease CRISPR-associated protein 9 (Cas9) that together function as an intra-cellular immune system providing resistance against foreign nucleic acids [16–18]. By co-expressing Cas9 and a single guide RNA (sgRNA) for a specific target gene, the CRISPR/Cas9 system can edit that specific gene through double-strand breaks (DSBs) and subsequent non-homologous end-joining (NHEJ) and homology-directed DNA repair mechanisms [19–21]. Thus, the CRISPR/cas9 system has been a powerful gene editing tool in biomedical research [22].

In this study, we targeted *miR-210-3p*, the most highly upregulated miRNA using the CRISPR/Cas9 system. Upon deleting *miR-210-3p* from multiple RCC cell lines, we surprisingly found that its downregulation resulted in increased tumorigenesis, both *in vitro* and *in vivo*. We also found that *miR-210-3p* downregulation resulted in dramatic upregulation of *TWIST1*. In addition, The Cancer Genome Atlas (TCGA) cohort analysis of ccRCC showed a negative correlation between *miR-210-3p* and *TWIST1* expression. Further, the low *miR-210-3p* and the high *TWIST1* expression groups had poor overall survival and disease free survival characteristics. Our data showed that *miR-210-3p* suppresses the expression of *TWIST1* that is required for progression of ccRCC. Our study also shows the novel application of the CRISPR/Cas9 system in cancer research to modulate the expression of miRNAs and other non-coding RNAs that are necessary for tumorigenesis and could potentially be used in cancer therapy.

## RESULTS

### *MiR-210-3p* is highly expressed in ccRCC tissues and RCC cell lines

**Figure 1:**
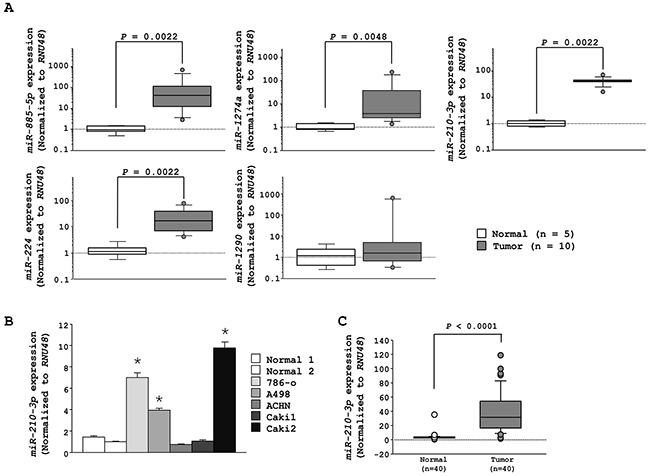
The expression levels of the top 5 upregulated miRNAs in RCC **A.** qRT-PCR data showing higher expression levels of *miR-885-5p*, *miR-1274a*, *miR-210-3p*, and *miR-224* in RCC tissues compared to adjacent noncancerous tissues (*p* = 0.0022, *p* = 0.0048, *p* = 0.0022 and *p* = 0.0022, respectively). **B.** qRT-PCR data showing high expression levels of *miR-210-3p* in 786-o, A498, and Caki2 RCC cell lines. **C.** qRT-PCR data showing high expression levels of *miR-210-3p* in RCC clinical tissues than in normal kidney samples (* *P* < 0.0001).

The 42 upregulated miRNAs (>2.5 fold) reported by our previous study in clinical ccRCC specimens (ten cancer tissues and five adjacent non-cancerous tissues) are shown in Table 1 [23]. The top five miRNAs (*miR-885-5p*, *miR-1274a*, *miR-210-3p*, *miR-224* and *miR-1290*) were re-analyzed using the same 15 clinical samples. Our analysis showed that of the five miRNAs, *miR-210-3p* was the most highly expressed (Figure 1A). Regarding the other four, the innate expression levels of *miR-885-5p* were found to be very low as represented by its Ct value; *miR-1274a* was shown to be part of ribosomal RNA [24]; and though the expression levels of *miR-224* were higher in the tumor tissues, it was ranked behind *miR-210-3p* (Table 1). Therefore, we focused on the role of *miR-210-3p* in ccRCC. Towards this, we found that *miR-210-3p* was highly expressed in three RCC cell lines (786-o, A498, and Caki2) compared to normal kidneys (*P* < 0.0001, Figure 1B). Further, we found that *miR-210-3p* was highly expressed in the ccRCC clinical specimens compared to the adjacent non-cancerous tissues (*n* = 40) (*P* < 0.0001, Figure 1C). The clinico-pathological information of the patients is shown in Supplementary Table 1. We observed that there was no significant relationship between any of the clinically relevant pathological parameters (i.e., tumor stage, grade, and survival rate) with the expression of *miR-210-3p* in the cohort of patients we analyzed (data not shown). These data suggested that the higher expression of *miR-210-3p* should be relevant for ccRCC progression.

**Table 1:** Up-regulated miRNAs in RCC samples

miRNA	ratio		Fold Change (T/N)
	Normal	Tumor	
hsa-miR-885-5p	1.03	68.69	66.62
hsa-miR-1274A	1.02	32.76	32.03
hsa-miR-210-3p	1.21	30.66	25.43
hsa-miR-224	1.06	23.55	22.14
hsa-miR-1290	1.47	28.73	19.49
hsa-miR-720	1.01	8.56	8.51
hsa-miR-592	1.15	7.43	6.48
hsa-miR-183	2.62	15.98	6.10
hsa-miR-1274B	1.04	5.41	5.20
hsa-miR-942	1.24	6.43	5.18
hsa-miR-15b	1.55	7.65	4.93
hsa-miR-155	1.18	5.73	4.86
hsa-miR-21	1.15	4.97	4.32
hsa-miR-486-3p	1.92	8.24	4.29
hsa-miR-130b	1.07	4.57	4.29
hsa-miR-642	1.23	5.11	4.17
hsa-miR-1300	1.38	5.70	4.13
hsa-miR-144	1.13	4.51	3.97
hsa-miR-1270	1.11	4.40	3.95
hsa-miR-21	1.18	4.60	3.90
hsa-miR-516-3p	1.59	5.84	3.67
hsa-miR-629	1.00	3.57	3.55
hsa-miR-616	1.01	3.57	3.53
hsa-miR-545	1.74	6.03	3.47
hsa-miR-1248	1.37	4.69	3.42
hsa-miR-1225-3P	1.24	4.25	3.42
hsa-miR-939	1.12	3.55	3.17
hsa-miR-34a	1.04	3.25	3.11
hsa-miR-629	1.04	3.19	3.08
hsa-miR-550	1.49	4.53	3.04
hsa-miR-452	1.34	4.01	2.99
hsa-miR-361-3p	1.32	3.81	2.89
hsa-miR-489	1.11	3.16	2.85
hsa-miR-34a	1.08	3.01	2.80
hsa-miR-589	1.29	3.58	2.77
hsa-miR-597	2.13	5.80	2.72
hsa-miR-193a-3p	1.19	3.16	2.65
hsa-miR-146b-5p	1.24	3.26	2.63
hsa-miR-451	1.18	3.07	2.60
hsa-miR-142-3p	1.24	3.13	2.52
hsa-miR-653	1.42	3.56	2.51
hsa-miR-142-5p	1.36	3.41	2.51

### Efficient knockdown of *miR-210-3p* in RCC cell lines by the CRISPR/Cas9 strategy

**Figure 2:**
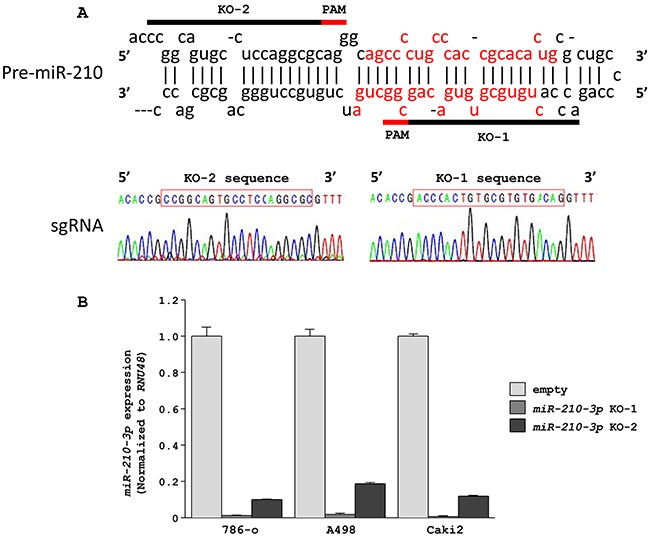
Downregulation of *miR-210-3p* in RCC cell lines using the CRISPR/Cas9 system **A**. Design of sgRNAs for *miR-210-3p*. Two sgRNAs were designed for *miR-210-3p* depletion by using CRISPR DESIGN (http://crispr.mit.edu/). Sequencing data showed that these sgRNAs were appropriately inserted into the lentiCRISPR v2 vector. **B**. qRT-PCR data showing that *miR-210-3p* was significantly depleted in the three RCC cell lines (786-o, A498, Caki2).

To investigate the functional role of *miR-210-3p* in ccRCC, we performed loss-of-function studies by using anti-*miR-210-3p* transfectants in the 786-o, A498, and Caki2 cell lines that highly expressed the *miR-210-3p*. However, only a 10% decrease in the expression of *miR-210-3p* was achieved although a high dose (30nM) of anti-*miR-210-3p* was used (Supplementary Figure 1). Therefore, to achieve high suppression of miR-210-3p (> 95%), we used the CRISPR/Cas9 system. We constructed two kinds of CRISPR/Cas9 vectors containing sgRNA designed for *miR-210-3p* using the CRISPR DESIGN tool (http://crispr.mit.edu/; Figure 2A). We confirmed proper insertion of each of the sgRNAs into the lenti-CRISPR v2 vector by direct sequencing (Figure 2A). Our analysis showed that the expression of *miR-210-3p* was significantly decreased in all 3 cell lines (786-o, A498 and Caki2) that were transfected with the *miR-210-3p* targeting CRISPR/Cas9 constructs in comparison to the empty vector controls. The knockdown was estimated to be >98% in all the three RCC cell lines showing the high efficiency of the CRISPR/Cas9 system (Figure 2B).

### Morphological and functional analysis of *miR-210-3p*-depleted ccRCC cells

**Figure 3:**
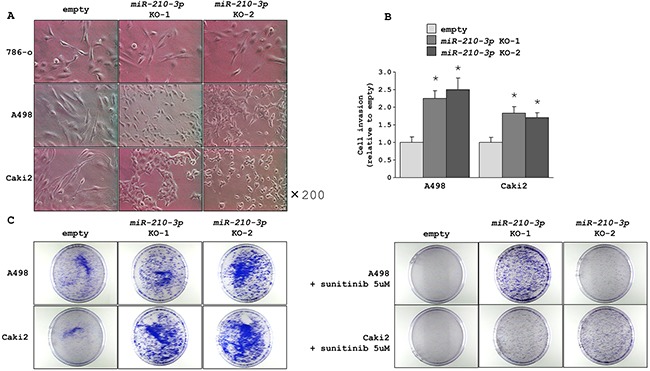
Morphological and functional characteristics of *miR-210-3p*-depleted ccRCC cells **A**. Representative images of *miR-210-3p*-depleted ccRCC cells showing morphological changes in the *miR-210-3p*-depleted A498 and Caki2 cells compared to the control. **B**. Matrigel invasion assay performed in A498 and Caki2 cell lines showing accelerated cell invasion in all the *miR-210-3p*-depleted cells (* *P* < 0.01). **C**. Representative images of colonies of A498 and Caki2 control cells and *miR-210-3p*-depleted cells with (right) or without (left) sunitinib malate.

We found that the A498 and Caki2 cell lines that had been depleted with *miR210-3p* showed dramatic morphological changes from a spindle to a round cell shape whereas no distinct changes were observed in the *miR-210-3p*-depleted 786-o cell line (Figure 3A). We postulated that the carrier mutations of *TP53* and *PTEN* in the 786-o cell line may have contributed to morphological differences observed between the three cell lines. To examine the effect of *miR-210-3p*-depletion on the RCC cell lines, we performed cell invasion assays using the A498 and Caki2 with depleted *miR-210-3p* and compared with the controls. We observed that the *miR-210-3p*-depleted cells showed accelerated cell invasiveness than the control cells (Figure 3B). In addition, colony formation assays using A498 and Caki2 with or without 5μM of sunitinib malate, a multi-targeted RTK inhibitor that is clinically used for RCC patients showed increased number of colonies in the *miR-210-3p*-depleted cells in comparison to the controls (Figure 3C). These results were surprising and suggested that the higher expression of *miR210-3p* found in the ccRCC clinical samples and the cell lines was probably inhibitory to tumor progression.

### Xenograft and ccRCC TCGA cohort studies to understand the *in vivo* effects of differential expression of *miR-210-3p*

**Figure 4:**
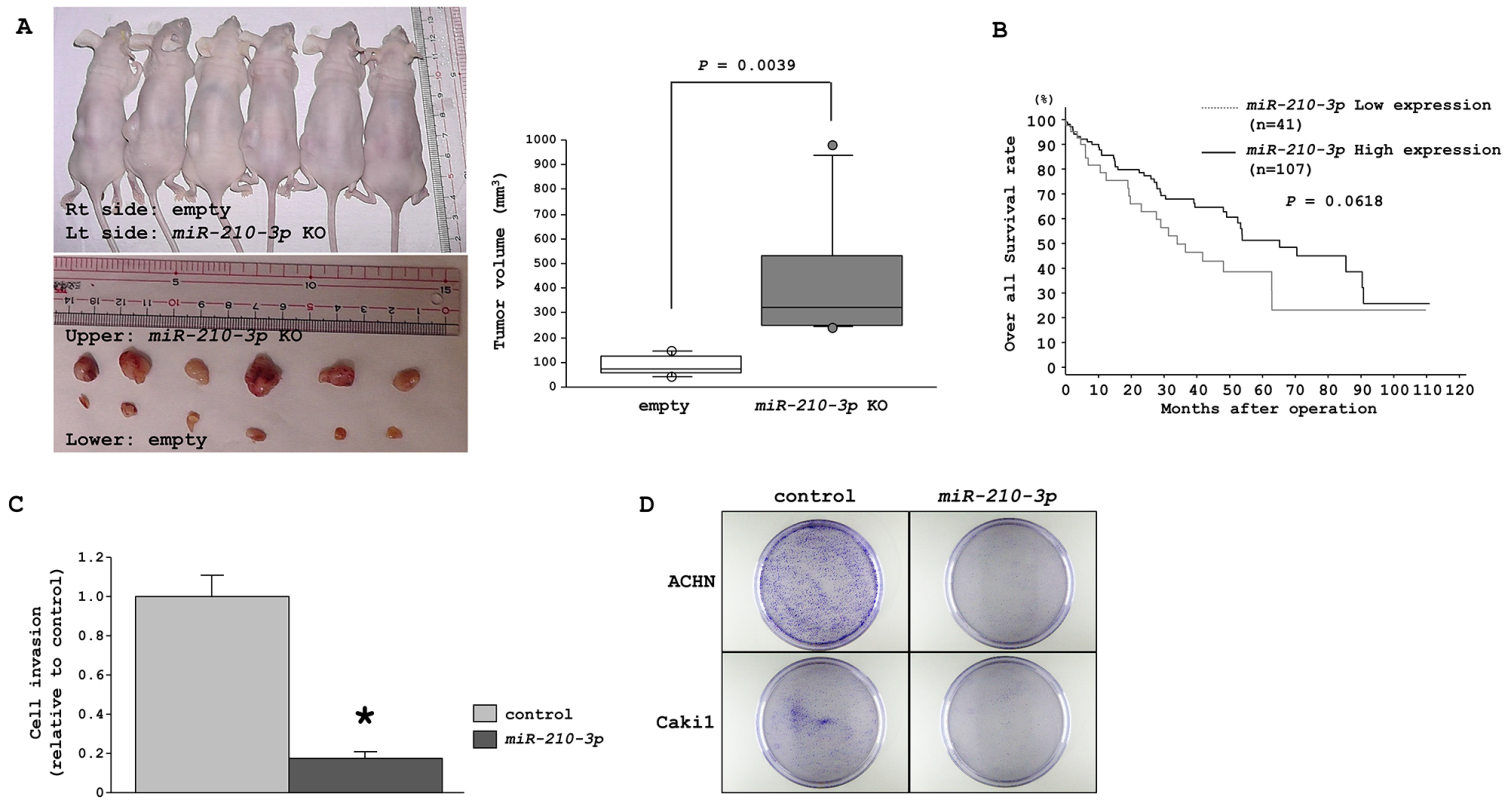
Characterization of tumor suppressive properties of *miR-210-3p* **A**. Representative images of the mice carrying tumors and the harvested tumors. Tumor volumes were calculated in nude mice 20 days after subcutaneous injection of A498 cells treated with *miR-210-3p*-depleted cells or with the empty vector (*P* = 0.0039). **B**. Patients with low *miR-210-3p* expression show reduced overall survival in comparison to those with high *miR-210-3p* expression (*P =0.0618*). **C**. Matrigel cell invasion assay performed with the ACHN cell line 72 h after *miR-210-3p* transfection (* *P* = 0.0003). **D**. Representative image of colony formation between miR-control and *miR-210-3p* transfectants in ACHN and Caki1 cells.

To evaluate the *in-vivo* effects of *miR-210-3p* expression levels on tumorigenicity, we injected *miR-210-3p*-depleted A498 cells subcutaneously into nude mice. A498 cell line was chosen over Caki2 because A498 cells had been extensively used in previous *in vivo* studies. We found that tumor growth was significantly enhanced in the mice xenografted with the *miR-210-3p* knock-out A498 cells (Figure 4A).

Further, we examined the *miR-210-3p* expression levels with patient prognosis using the TCGA database. Based on median value of *miR210-3p* expression, the cohort divided into two groups, namely, a low *miR-210-3p* expression group (n = 41) that showed poor median survival of 33.97 months in comparison with the high *miR-210-3p* expression group (n = 107) that showed higher median survival of 65.22 months (*P* = 0.0618, Figure 4B). These results confirmed that *miR-210-3p* expression plays an important role in RCC progression *in vivo*.

### Effects of enhancing *miR-210-3p* expression on cellular migration and colony formation

We investigated the effects of enhancing *miR-210-3p* expression using miRNA transfection in *miR-210-3p* low expressing cell lines, ACHN and Caki1. The matrigel invasion assays showed that enhancing *miR210-3p* expression reduced cell invasiveness in the ACHN cell line (*P* = 0.0003, Figure 4C), whereas Caki1 did not get into the matrigel chamber and hence the data could not be analyzed. In colony formation assay, we observed decreased number of colonies in *miR-210-3p* overexpressed cells in comparison to the control cells (Figure 4D). This suggested that *miR-210-3p* overexpression had a suppressive effect on cell invasiveness and colony growth characteristics of RCC cell lines

### Identification of targets genes regulated by *miR-210-3p* in ccRCC

**Figure 5:**
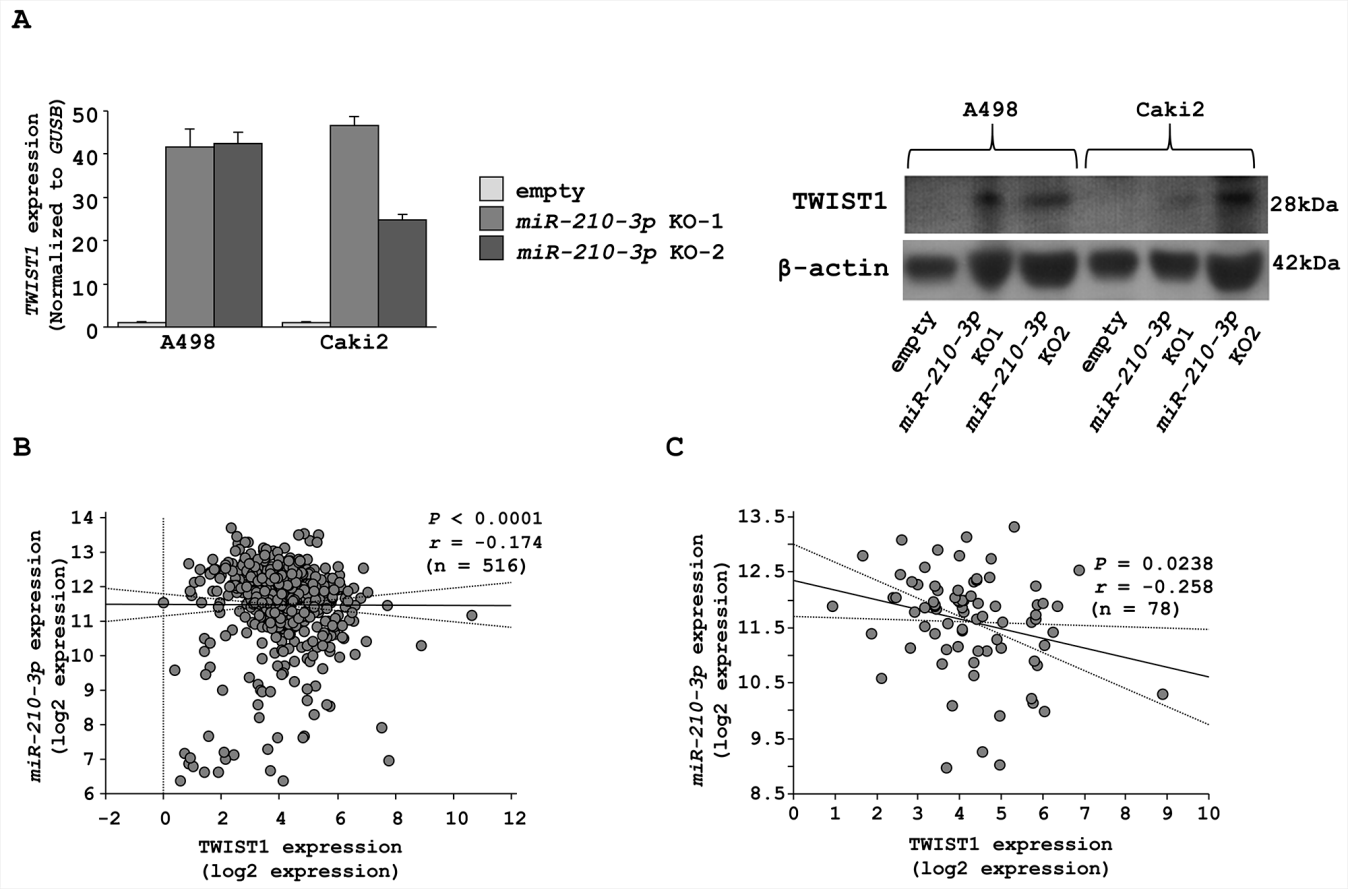
Characterization of *TWIST1* as a candidate target of *miR-210-3p* **A**. *TWIST1* mRNA and protein expression in *miR-210-3p*-depleted A498 and Caki2 cell lines. **B**. Correlation between *miR-210-3p* and *TWIST1* mRNA expression in clinical RCC patients. Correlation between *miR-210-3p* and *TWIST1* mRNA expression in RCC patients with metastasis. Gene expression values were estimated using RSEM.

Having identified the functional phenotype of *miR-210-3p* expression in ccRCC, we further wanted to identify its target genes to further gain insights into the molecular mechanism of *miR-210-3p* in RCC. Towards this, we used multiple databases to identify the target genes including, the GEO databases (GSE36895 and GSE22541), the TargetScan and the TCGA. Our strategy involved identifying the upregulated genes in the RCCs using the GEO datasets, followed by selecting the genes with *miR-210-3p* target site in their 3′ -untranslated region (UTR) as analyzed using the TargetScan database and finally checking their clinical relevance using TCGA. Based on this approach, we identified eight candidate genes (*ADH5*, *FBXO21*, *KCNJ15*, *KIF13B*, *SDHD*, *SERPINA3*, *TPRG1L*, *and TWIST1*) that are listed in Supplementary Table 2. Among the eight genes, we focused on *TWIST1* that regulates epithelial-mesenchymal transition (EMT) since we had observed morphological transitioning in the *miR-210-3p* knock-out cells. As expected, qRT-PCR showed that *TWIST1* mRNA expression was dramatically increased in *miR-210-3p*-depleted A498 and Caki2 cells (Figure 5A left, Supplementary Figure 2). This data was supported by western blotting analysis that confirmed that the TWIST1 protein expression was increased in *miR-210-3p*-depleted A498 and Caki2 cell lines (Figure 5A Right).

Further, we investigated the relationship between *miR-210-3p* and *TWIST1* expression levels in the RCC patient samples using the TCGA database. We observed a negative correlation between *miR-210-3p* and the expression of *TWIST1* mRNA according to the Spearman's rank test (Figure 5B). Also, there was a stronger negative correlation between *miR-210-3p* and *TWIST1* mRNA in RCC patients with metastasis (r = -0.258) in comparison to all RCC patients (r = -0.174) as shown in Figure 5C.

**Figure 6:**
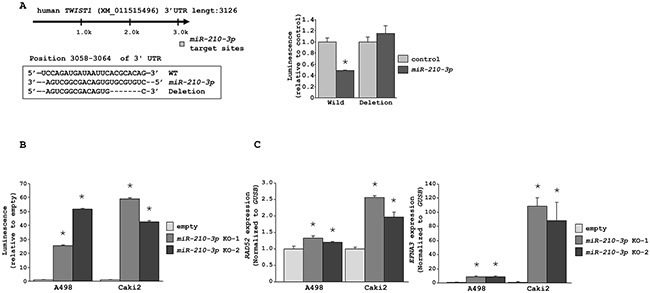
*miR-210-3p* directly regulates *TWIST1* **A**. Luciferase reporter assays using two vectors encoding either putative *miR-210-3p* binding site in the 3′-UTR of *TWIST1* (nucleotides 3058-3064) or after deleting the *miR-210-3p* binding site. Luminescence intensity was measured in the *miR-210-3p* transfectants in comparison with the miR-control transfectant. *Renilla* luciferase values were normalized to firefly luciferase values (* *P* = 0.0495). **B**. Luciferase reporter assays using the vector encoding *TWIST1* 3′-UTR in *miR-210-3p*-depleted A498 and Caki2 cell lines (* *P* < 0.0001). **C**. qRT-PCR data showing that the expression levels of *RAD52* and *EFNA3*, known targets of *miR-210-3p*, were significantly higher in *miR-210-3p*-depleted A498 and Caki2 cell lines (* *P* < 0.0001).

To confirm the link between *miR-210-3p* and *TWIST1*, we performed luciferase reporter assays to determine whether the 3′-UTR of *TWIST1* contained an actual binding site for *miR-210-3p*. The Targetscan database predicted a single putative *miR-210-3p* binding site in the 3′-UTR of *TWIST1* (positions 3058-3064 in XM_011515496; Figure 6A). Our data showed that the luminescence intensity from the wild-type vector carrying the 3′-UTR of *TWIST1* was significantly reduced by *miR-210-3p* transfection, whereas the deletion vector showed no change in luminescence upon *miR-210-3p* transfection (*P* < 0.0495; Figure 6A). When similar luciferase reporter assays were performed using the vector encoding *TWIST1* 3′-UTR in *miR-210-3p*-depleted A498 and Caki2 cell lines, the luminescence intensity increased greatly in *miR-210-3p*-depleted A498 and Caki2 cell lines (* P < 0.0001, Figure 6B). Further, we observed increased expression levels of *RAD52* and *EFNA3* that are known targets of *miR-210-3p* [25, 26], in the A498 and Caki2 cell lines depleted with *miR-210-3p* as analyzed by qRT-PCR (* P < 0.0001, Figure 6C).

### *TWIST1* upregulation correlates with poor overall survival and shortened disease-free survival in ccRCC

**Figure 7:**
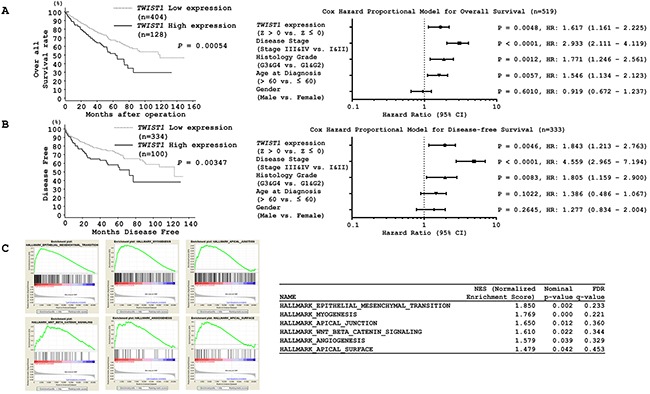
Kaplan-Meier survival plots for high and low *TWIST1* expression groups in a TCGA cohort **A**. Overall survival and **B**. Disease-free survival periods (left) were significantly reduced in the patients with high *TWIST1* expression in comparison with the patients with low expression (*P =0.00054* and *P = 0.00347* respectively). (A) Cox proportional analysis for the prediction of overall survival or (B, left) disease-free survival. **C**. GSEA indicating six significantly enriched pathways, of which EMT was the most significant.

We then evaluated the correlation of *TWIST1* expression levels with prognosis and disease-free survival of ccRCC patients using the TCGA database. The cohort was divided into two groups based on the median value. We found that the high *TWIST1* expression group (n = 128) had poor median overall survival of 62.84 months in comparison with the low *TWIST1* expression group (n = 404) that had a median overall survival of 118.76 months (*P* = 0.00054, Figure 7A left). A similar effect was found for disease-free survival. The high *TWIST1* expression group (n = 100) had a median disease free survival of 72.9 months in comparison to the low *TWIST1* expression group (n = 334) that had a median disease free survival of 123.72 months (*P* = 0.00347, Figure 7B left). The multivariate Cox proportional hazards model showed that *TWIST1* expression was an independent predictor of overall survival and disease-free survival. Also, *TWIST1* expression was an independent predictor when analyzed for disease stage (Stage III&IV vs. Stage I&II) and histology grade (G3&G4 vs. G1&G2) parameters (Figures 7A right and 7B right). Furthermore, GSEA (gene set enrichment analysis) data comparing the *TWIST1* high and low expression groups identified six enriched pathways of which EMT was the most significantly enriched pathway (Figure 7C). All these data put together strongly suggest that *TWIST1* elevation by *miR-210-3p* depletion promotes EMT and results in poor outcomes for ccRCC patients.

## DISCUSSION

Although several studies have successfully used miRNA inhibitors, morpholinos or sponges to downregulate miRNA expression the knock down efficiency has not been fully satisfactory with off-target effects [11–13]. Also, although upregulated miRNAs have been used as markers [14], the functional insights have not been well understood. Three methods namely, zinc-finger nucleases (ZFNs), transcription activator-like effector nucleases (TALENs) and the CRISPR/Cas9 system have been used to perform gene editing in a variety of *in vitro* and *in vivo* models [27]. Since CRISPR/Cas9 system is more convenient, flexible, and powerful, it has been preferred over ZFN and TALEN for gene editing. Whereas DNA editing has been performed for protein coding genes its utility for non-coding RNAs has not been fully explored. Few recent studies have used the CRISPR/Cas9 system to downregulate miRNA expression in human cells [28–30]. Jiang and colleagues successfully knocked out human *miR-93* by targeting its 5′ region [28]. Chang and others targeted 3′-UTR of their target miRNAs with a lenti-CRISPR vector, and depleted their target miRNAs up to 96% [29]. We used the same methodology as used by Chang and colleagues except that a newer version of lentivirus vector that can produce 10-fold higher viral titers was used to get better knockdown efficiency of greater than 98%. Because precursor miRNA are no longer than 100 bases, the candidate sequences of sgRNAs are limited. There are two versions of *miR-210*, namely, *miR-210-3p* and *miR210-5p*. *miR-210-3p* is the guide-strand that integrates into the RNA induced silencing complex (RISC), whereas *miR-210-5p* is the passenger-strand that is inactivated through degradation [6]. For our studies, we designed two sgRNAs for *miR-210*, one of within the 3p and the second within the 5p. The knockdown efficiency in cells induced with sgRNA targeting *miR-210-3p* was more than 98%, whereas the knockdown efficiency for *miR-210-5p* was between 81 and 90%. The differences in the knockdown efficiency probably reflected on the sgRNA target sites. Both the knockdown cells showed a common morphological phenotype (spindle shaped to rounded cell shape change) that was not observed in previous experiments with anti-miRNA where the knockdown was not substantial. These changes were not off-target effects of the CRISPR/Cas9 system because they were robustly observed in both the A498 and Caki2 cell lines that were transfected with the two different sgRNAs. However, the 786-o knockdown cells showed no changes in cell morphology and TWIST1 expression in spite of significant knockdown of *miR-210-3p*. This probably reflected the effects of the inherent *TP53* and *PTEN* mutations in the 786-o cells. Several studies have demonstrated that *TP53* or *PTEN* mutations acquire resistance to chemotherapy or display neomorphic activity that is independent of its phosphatase activity [31–33]. Further examination is necessary to elucidate the morphological and TWIST1 expression discrepancy between 786-o and A498/Caki2. Furthermore, there were no cell morphology changes between *TWIST1*-overxepressed A498/Caki2 cells and *TWIST1*-knockdown *miR-210-3p*-depleted A498/Caki2 cells (data not shown), suggesting that *miR-210-3p* may regulate other EMT-related genes either directly or indirectly apart from *TWIST1*. Also, it showed that *TWIST1* was not solely responsible for the morphological changes observed upon *miR-210-3p* knockdown in ccRCC cells.

In this study, we focused on the functional significance of the upregulated *miR-210-3p* in ccRCC. Depletion of *miR-210-3p* significantly increased cell invasiveness *in vitro* and promoted tumorigenesis in xenograft experiments *in vivo*. McCormick and others have reported that *miR-210-3p* was generally upregulated in clinical ccRCC tissues compared to normal tissues [34]. However, its expression was lower in high grade and late stage ccRCC in comparison with low grade and early stage [34]. In addition, lower expression of *miR-210-3p* showed high Ki-67 expression, which is activated during advanced stages of cancer and correlated with poor survival. Our data from the *in vitro* and *in vivo* experiments are consistent with these studies. In addition, *miR-210-3p* gain-of-function studies in ACHN and Caki1 cells indicated tumor suppressive function. We postulate that *miR-210-3p* expression has dual consequences in tumorigenesis and metastasis. Upregulation of *miR-210-3p* may be necessary to establish tumorigenesis in ccRCC. However, in order to achieve EMT and metastasize, *miR-210-3p* needs to be downregulated in order to release its suppression of *TWIST1*. This maybe the reason why *miR-210-3p* is found to be downregulated in high grade and late stage ccRCC compared to low grade and early stage ccRCC.

Although multiple miRNAs (*miR-33a*, *miR-106a/b* and *miR-214*) have been reported to regulate *TWIST1* [35], this is the first study to show that *miR-210-3p* directly regulates *TWIST1* expression. Also, the TCGA cohort analysis showed a negative correlation between the expression of *TWIST1* and *miR-210-3p*. Consequently, the high *TWIST1* expression group showed poor overall survival and shorter disease-free survival in comparison with the low expression group. TWIST1 is known to have important roles in some physiological processes involved in metastasis and invasion through EMT regulation [36]. Also, TWIST1 has been shown to be involved in evading apoptosis [36]. In this study, we demonstrated that cell invasion was accelerated *in vitro* and tumor growth was increased when *miR-210-3p* depleted cells were xenografted. However, due to experimental limitations we could not perform the *in vivo* imaging experiments and the apoptosis assay to determine further mechanistic details underlying the role of *miR-210-3p*. However, we postulate that *miR-210-3p* levels determine cancer cell migration and/or invasion due to its effects on *TWIST1* that promotes EMT. In addition, we did not demonstrate the mechanism by which *miR-210-3p* expression was decreased in advanced RCC samples. The key factor could be hypoxia inducible factor (HIF) because HIF1 is known to regulate *miR-210-3p* as well as TWIST1 [37, 38]. Therefore, further studies are necessary to determine the regulation of *miR-210-3p*. Also, we have successfully demonstrated the use of the CRISPR/Cas9 system to investigate oncogenic pathways associated with upregulated miRNAs. Previously, few studies had used the CRISPR/Cas9 system to downregulate miRNAs expression in cancer cells [29, 30], but did not pursue detailed analyses. Therefore, this might be the first paper to use the CRISPR/Cas9 system to demonstrate an oncogenic mechanism associated with upregulated miRNA.

In summary, we demonstrate the utility of the CRISPR/Cas9 system to study specific miRNAs and other non-coding RNAs in areas of cancer research by successfully downregulating *miR-210-3p* expression in RCC cells. We further demonstrated that progression in tumorigenesis into metastatic phase in ccRCC was acquired through enhanced expression of oncogenic TWIST1 when *miR-210-3p* expression was downregulated.

## MATERIALS AND METHODS

### Clinical specimens and cell culture

Following nephrectomies at Kagoshima University Hospital, five pairs of clear cell type cancer and adjacent non-cancerous tissues and an additional group of five clear cell type cancer tissues were collected for miRNA expression analysis [23]. For qRT-PCR, tissue specimens were obtained from 40 ccRCC patients who had also undergone nephrectomies at Kagoshima University Hospital between 2005 and 2010 (Supplementary Table 1). The samples were staged according to American Joint Committee on Cancer-Union Internationale Contre le Cancer (AJCC-UICC) classification and histologically graded [Wiley-Liss; 2009, p.255-257]. Our study was approved by the Bioethics Committee of Kagoshima University and written prior informed consent and approval was obtained from all patients. We used five human RCC cell lines: 786-o, A498, ACHN, Caki1, and Caki2 that were obtained from the American Type Culture Collection (ATCC; Manassas, VA, USA). These cell lines were grown in RPMI 1640 medium (Invitrogen; Carlsbad, CA, USA) supplemented with 10% fetal bovine serum (FBS) and maintained in humidified incubators (5% CO_2_) at 37°C.

### Tissue collection and RNA extraction

Tissues were stored in RNAlater (Thermo Fisher Scientific; Waltham, Massachusetts, USA) and maintained at -20°C until RNA extraction. Total RNA including miRNA was extracted using the mirVana miRNA isolation kit (Ambion; Austin, TX, USA) according to manufacturer's protocol. The integrity of the RNA was checked with the RNA 6000 Nano Assay Kit in a 2100 Bioanalyzer (Agilent Technologies; Santa Clara, CA, USA).

### Quantitative real-time RT-PCR (qRT-PCR)

The procedure for PCR quantification was as described previously [39]. The expression levels of *miR-885-5p* (Assay ID: 002296), *miR-1274a* (Assay ID: 002883), *miR-210-3p* (Assay ID: 000512), *miR-224* (Assay ID: 002099) and *miR-1290* (Assay ID: 002863) were analyzed by TaqMan qRT-PCR (TaqMan MicroRNA Assay; Thermo Fisher Scientific) and normalised to RNU48 (P/N: 001006; Thermo Fisher Scientific). The delta–delta Ct method was employed to calculate the fold-change. For the protein coding genes, SYBR-green quantitative PCR-based array approach was used and the sequences are listed in Supplementary Table 2. qRT-PCR was performed with 500 ng of total RNA using the Power SYBR Green Master Mix (cat. no. 4367659; Applied Biosystems, Foster City, CA) on the 7300 Real-time PCR System (Applied Biosystems). The specificity of amplification was monitored by the dissociation curve of the amplified product. Fold changes were calculated after normalizing the data to that of *GUSB* and applying the delta–delta Ct method. For control RNA, total RNA from two normal human kidneys was used (AM 7976; Applied Biosystems. cat. no. 636529; Clontech, Mountain View, CA).

### Transfection with anti-miRNA and mature miRNA

RCC cell lines were transfected with 10-60nM anti-miRNA or 10nM mature miRNA, Lipofectamine RNAiMAX transfection reagent (Thermo Fisher Scientific) and Opti-MEM (Thermo Fisher Scientific) as previously described [39]. For miRNA loss of function experiments, anti- miRNA Inhibitor negative Control (product ID: PM17010; Thermo Fisher Scientific) and hsa-anti-*miR-210-3p* (product ID: PM17000) were used. For miRNA gain of function experiments, negative-control miRNA (product ID: PM17111; Thermo Fisher Scientific) and mature *miR-210-3p* (product ID: PM17100; Thermo Fisher Scientific) were used.

### Lentivirus production and transduction

The high titer virus producing lenti-CRISPR plasmid vector was a gift from Dr. Feng Zhang's lab through Addgene (#52961) [40]. The sgRNAs targeting *miR-210-3p* were designed by CRISPR DESIGN (http://crispr.mit.edu/) and set just before the protospacer adjacent motif (PAM), a DNA sequence immediately following the Cas9 targeted DNA sequence. All the specific target sequences were amplified and cloned into lenti-CRISPR vectors and verified by DNA sequencing. To produce lentivirus, HEK 293T cells seeded in 100-mm plates were transfected with 4.0μg lenti-CRISPR-v2 plasmids, 3.0μg psPAX2 and 1.0μg pMD2G plasmids using polyethyleneimine reagent (Polysciences; Warrington, PA, USA) according to the manufacturer's instructions. HEK 293T cells were grown in DMEM (ATCC) containing 10% FBS for 72 h following which the supernatants of the transfected cells containing lentivirus were harvested, passed through a 0.45micron filter and stored at − 80 °C. For transduction with lentivirus, the 786-o, A498 and Caki2 cells (2 × 10^5^) were seeded in 6-well plates and the spin-transduction was performed by centrifuging the plate coated with 8 μ g/mL polybrene (sc-134220, Santa Cruz Biotechnology; Dallas, TX, USA) at 2600 rpm for 20 min at 25°C. The cells were cultured for 12 h followed by adding fresh RPMI 1640 medium supplemented with 10% FBS and treated for six days with 0.5ug/mL puromycin (sc-108071, Santa Cruz Biotechnology) for selection. The puromycin selected cells were then expanded in regular culture medium (RPMI 1640 with 10% FBS).

### Cell invasion assays

The cell invasion assays were performed using modified Boyden chambers that contain transwell-precoated matrigel membrane filter inserts with 8μm pores in 24-well tissue culture plates (BD Biosciences, Bedford, MA, USA). A498 and Caki2 cell lines that were either depleted of *miR-210-3p* as confirmed by qRT-PCR or transfected by mature *miR-210-3p* for 48h were seeded in 24-well plates (1×10^5^ cells/well). RPMI 1640 containing 10% FBS was added in the lower chamber as a chemoattractant. All experiments were performed in triplicate according to manufacturer's instructions.

### Colony formation assays

For colony formation assays to test the effect of the multi-RTK inhibitor, 5000 cells were plated into a 10cm dish and cultured with or without 5uM of Sunitinib Malate (Selleck Chemicals, Cat. No. S1042) for 6 days to allow optimal colony formation followed by staining with 0.04% crystal violet (Nacalai tesque, Kyoto, Japan).

### Western blot analysis

Thirty micrograms of total cellular protein was separated by NuPAGE on 4–12% Bis–Tris gels (Invitrogen) and transferred onto PVDF membranes. Immunoblotting was carried out with diluted (1:1000) anti-TWIST1 antibodies (sc-15393, Santa Cruz Biotechnology) and anti-β-actin antibodies (bs-0061R, Bioss; Woburn, MA, USA). The blots were visualised with ECL detection system (GE Healthcare, Little Chalfont, UK).

### Plasmid construction and dual-luciferase reporter assays

A partial wild-type sequence of the *TWIST1* 3′ UTR or the same sequence with the deleted *miR-210-3p* target site (positions 3058–3064 of the *TWIST1* 3′-UTR, according to the TargetScan program (http://www.targetscan.org/)) was inserted between the XhoI and PmeI restriction sites in the 3′-UTR of the *hRluc* gene in the psi-CHECK-2 vector (C8021, Promega; Madison, WI, USA). A498 and Caki2 cells were transfected with 5ng of the vector and 10nM *miR-210-3p* or control miRNAs using Lipofectamine 2000 (Invitrogen). For another set of experiments, *miR-210-3p* knocked-out A498 and Caki2 cells were transfected with 5ng of the vector encoding a partial wild-type sequence of the *TWIST1* 3′-UTR. The activities of firefly and *Renilla* luciferases in cell lysates were determined with a dual-luciferase assay system (E1960, Promega). Normalized data was calculated as the ratio of *Renilla* /firefly luciferase activities.

### *In vivo* tumor xenograft model

To confirm *miR-210-3p* function *in vivo*, a mixture containing100μL A498 cells (4 × 10^6^) that are depleted of *miR-210-3p* or control A498 cells with empty lenti CRISPR v2 vector and 100μL Matrigel Matrix (Corning; Corning, Bedford, MA, USA) was injected subcutaneously into the right and left flanks of 6 female nude mice (BALB/c nu/nu, 6- to 8-week-old). The tumor sizes and weights were measured twice a week and tumor volumes were calculated [(tumor length × width x 2)/2]. Twenty days after inoculation, all the mice were sacrificed and their tumor masses were excised, measured and photographed. All the animal experiments were performed in accordance with institutional guidelines and were approved by the animal care review board of Kagoshima University.

### Analysis of overall survival of a ccRCC cohort from The Cancer Genome Atlas (TCGA)

The TCGA cohort database was used to determine the relationship between *TWIST1* expression and overall survival. RSEM was used for gene expression quantification [41]. mRNA expression Z-scores by whole-exome sequencing data were available for 538 ccRCC patients. Full sequencing information and clinical information was acquired from the cBio Portal (http://www.cbioportal.org/public-portal/) and the TCGA (https://cancergenome.nih.gov/) [42–44]. GSEA was performed to identify enriched pathways using open source software v2.0 (www.broad.mit.edu).

### Statistical analysis

The statistical relationship between two groups was analysed using the Mann-Whitney *U* test. The relationships between three variables and numerical values were analysed using Bonferroni-adjusted Mann-Whitney *U* test. Spearman's rank test was used to evaluate the correlation between the expression of *miR-210-3p* and *TWIST1*. Overall survival of ccRCC patients from the TCGA cohort was evaluated by the Kaplan-Meier method. Patients were divided into two groups based on the median value of *TWIST1* expression, and the differences between the two groups were evaluated by the log-rank tests. A multivariate Cox proportional hazards model was used to establish independent factors for overall survival. The chi square test was used to analyze the relationship between *TWIST1* expression and the various clinico-pathological characteristics. We used Expert Stat View software, version 5.0 (Cary, NC, USA) was used for these analyses.

## SUPPLEMENTARY MATERIALS FIGURES AND TABLES



## References

[1] Hadoux J, Vignot S, De La Motte Rouge T (2010). Renal cell carcinoma: focus on safety and efficacy of temsirolimus. Clin Med Insights Oncol.

[2] Gupta K, Miller JD, Li JZ, Russell MW, Charbonneau C (2008). Epidemiologic and socioeconomic burden of metastatic renal cell carcinoma (mRCC): a literature review. Cancer Treat Rev.

[3] Naito S, Tomita Y, Rha SY, Uemura H, Oya M, Song HZ, Zhong LH, Wahid MI (2010). Kidney Cancer Working Group report. Jpn J Clin Oncol.

[4] Sharma P, Allison JP (2015). Immune checkpoint targeting in cancer therapy: toward combination strategies with curative potential. Cell.

[5] Filipowicz W, Bhattacharyya SN, Sonenberg N (2008). Mechanisms of post-transcriptional regulation by microRNAs: are the answers in sight?. Nat Rev Genet.

[6] Bartel DP (2004). MicroRNAs: genomics, biogenesis, mechanism, and function. Cell.

[7] Nelson KM, Weiss GJ (2008). MicroRNAs and cancer: past, present, and potential future. Mol Cancer Ther.

[8] Lin S, Gregory RI (2015). MicroRNA biogenesis pathways in cancer. Nat Rev Cancer.

[9] Yoshino H, Seki N, Itesako T, Chiyomaru T, Nakagawa M, Enokida H (2013). Aberrant expression of microRNAs in bladder cancer. Nat Rev Urol.

[10] Yoshino H, Enokida H, Itesako T, Kojima S, Kinoshita T, Tatarano S, Chiyomaru T, Nakagawa M, Seki N (2013). Tumor-suppressive microRNA-143/145 cluster targets hexokinase-2 in renal cell carcinoma. Cancer Sci.

[11] Stenvang J, Petri A, Lindow M, Obad S, Kauppinen S (2012). Inhibition of microRNA function by antimiR oligonucleotides. Silence.

[12] Kloosterman WP, Lagendijk AK, Ketting RF, Moulton JD, Plasterk RH (2007). Targeted inhibition of miRNA maturation with morpholinos reveals a role for miR-375 in pancreatic islet development. PLoS Biol.

[13] Ebert MS, Neilson JR, Sharp PA (2007). MicroRNA sponges: competitive inhibitors of small RNAs in mammalian cells. Nat Methods.

[14] Iorio MV, Croce CM (2012). MicroRNA dysregulation in cancer: diagnostics, monitoring and therapeutics. A comprehensive review. EMBO Mol Med.

[15] Ishino Y, Shinagawa H, Makino K, Amemura M, Nakata A (1987). Nucleotide sequence of the iap gene, responsible for alkaline phosphatase isozyme conversion in Escherichia coli, and identification of the gene product. J Bacteriol.

[16] Brouns SJ, Jore MM, Lundgren M, Westra ER, Slijkhuis RJ, Snijders AP, Dickman MJ, Makarova KS, Koonin EV, van der Oost J (2008). Small CRISPR RNAs guide antiviral defense in prokaryotes. Science.

[17] Gasiunas G, Barrangou R, Horvath P, Siksnys V (2012). Cas9-crRNA ribonucleoprotein complex mediates specific DNA cleavage for adaptive immunity in bacteria. Proc Natl Acad Sci U S A.

[18] Jinek M, Chylinski K, Fonfara I, Hauer M, Doudna JA, Charpentier E (2012). A programmable dual-RNA-guided DNA endonuclease in adaptive bacterial immunity. Science.

[19] Gratz SJ, Cummings AM, Nguyen JN, Hamm DC, Donohue LK, Harrison MM, Wildonger J, O’Connor-Giles KM (2013). Genome engineering of Drosophila with the CRISPR RNA-guided Cas9 nuclease. Genetics.

[20] Wang H, Yang H, Shivalila CS, Dawlaty MM, Cheng AW, Zhang F, Jaenisch R (2013). One-step generation of mice carrying mutations in multiple genes by CRISPR/Cas-mediated genome engineering. Cell.

[21] Zhang F, Wen Y, Guo X (2014). CRISPR/Cas9 for genome editing: progress, implications and challenges. Hum Mol Genet.

[22] Hsu PD, Lander ES, Zhang F (2014). Development and applications of CRISPR-Cas9 for genome engineering. Cell.

[23] Hidaka H, Seki N, Yoshino H, Yamasaki T, Yamada Y, Nohata N, Fuse M, Nakagawa M, Enokida H (2012). Tumor suppressive microRNA-1285 regulates novel molecular targets: aberrant expression and functional significance in renal cell carcinoma. Oncotarget.

[24] Schopman NC, Heynen S, Haasnoot J, Berkhout B (2010). A miRNA-tRNA mix-up: tRNA origin of proposed miRNA. RNA Biol.

[25] Crosby ME, Kulshreshtha R, Ivan M, Glazer PM (2009). MicroRNA regulation of DNA repair gene expression in hypoxic stress. Cancer Res.

[26] Hu YW, Jiang JJ, Yan G, Wang RY, Tu GJ (2016). MicroRNA-210 promotes sensory axon regeneration of adult mice in vivo and in vitro. Neurosci lett.

[27] Gaj T, Gersbach CA, Barbas CF, ZFN 3rd (2013). TALEN and CRISPR/Cas-based methods for genome engineering. Trends Biotechnol.

[28] Jiang Q, Meng X, Meng L, Chang N, Xiong J, Cao H, Liang Z (2014). Small indels induced by CRISPR/Cas9 in the 5′ region of microRNA lead to its depletion and Drosha processing retardance. RNA Biol.

[29] Chang H, Yi B, Ma R, Zhang X, Zhao H, Xi Y (2016). CRISPR/cas9, a novel genomic tool to knock down microRNA in vitro and in vivo. Sci Rep.

[30] Ho TT, Zhou N, Huang J, Koirala P, Xu M, Fung R, Wu F, Mo YY (2015). Targeting non-coding RNAs with the CRISPR/Cas9 system in human cell lines. Nucleic Acids Res.

[31] Blandino G, Levine AJ, Oren M (1999). Mutant p53 gain of function: differential effects of different p53 mutants on resistance of cultured cells to chemotherapy. Oncogene.

[32] Fernandez S, Genis L, Torres-Aleman I (2014). A phosphatase-independent gain-of-function mutation in PTEN triggers aberrant cell growth in astrocytes through an autocrine IGF-1 loop. Oncogene.

[33] Memmel S, Sukhorukov VL, Horing M, Westerling K, Fiedler V, Katzer A, Krohne G, Flentje M, Djuzenova CS (2014). Cell surface area and membrane folding in glioblastoma cell lines differing in PTEN and p53 status. PloS one.

[34] McCormick RI, Blick C, Ragoussis J, Schoedel J, Mole DR, Young AC, Selby PJ, Banks RE, Harris AL (2013). miR-210 is a target of hypoxia-inducible factors 1 and 2 in renal cancer, regulates ISCU and correlates with good prognosis. Br J Cancer.

[35] Khanbabaei H, Teimoori A, Mohammadi M (2016). The interplay between microRNAs and Twist1 transcription factor: a systematic review. Tumour biology.

[36] Puisieux A, Valsesia-Wittmann S, Ansieau S (2006). A twist for survival and cancer progression. Br J Cancer.

[37] Crosby ME, Kulshreshtha R, Ivan M, Glazer PM (2009). MicroRNA regulation of DNA repair gene expression in hypoxic stress. Cancer Res.

[38] Yang MH, Wu MZ, Chiou SH, Chen PM, Chang SY, Liu CJ, Teng SC, Wu KJ (2008). Direct regulation of TWIST by HIF-1alpha promotes metastasis. Nat Cell Biol.

[39] Nohata N, Hanazawa T, Kinoshita T, Inamine A, Kikkawa N, Itesako T, Yoshino H, Enokida H, Nakagawa M, Okamoto Y, Seki N (2013). Tumour-suppressive microRNA-874 contributes to cell proliferation through targeting of histone deacetylase 1 in head and neck squamous cell carcinoma. Br J Cancer.

[40] Sanjana NE, Shalem O, Zhang F (2014). Improved vectors and genome-wide libraries for CRISPR screening. Nat Methods.

[41] Li B, Dewey CN (2011). RSEM: accurate transcript quantification from RNA-Seq data with or without a reference genome. BMC bioinformatics.

[42] Gao J, Aksoy BA, Dogrusoz U, Dresdner G, Gross B, Sumer SO, Sun Y, Jacobsen A, Sinha R, Larsson E, Cerami E, Sander C, Schultz N (2013). Integrative analysis of complex cancer genomics and clinical profiles using the cBioPortal. Sci Signal.

[43] Cerami E, Gao J, Dogrusoz U, Gross BE, Sumer SO, Aksoy BA, Jacobsen A, Byrne CJ, Heuer ML, Larsson E, Antipin Y, Reva B, Goldberg AP, Sander C, Schultz N (2012). The cBio cancer genomics portal: an open platform for exploring multidimensional cancer genomics data. Cancer Discov.

[44] Cancer Genome Atlas Research N (2013). Comprehensive molecular characterization of clear cell renal cell carcinoma. Nature.

