# Supplementary material for: microRNA-210-3p depletion by CRISPR/Cas9 promoted tumorigenesis through revival of TWIST1 in renal cell carcinoma
Source: Oncotarget. 2017 Feb 1;8(13):20881–94. doi: 10.18632/oncotarget.14930 (PMC5400553; doi:10.18632/oncotarget.14930)
Supplement: Supplementary file 1 [file oncotarget-08-20881-s001.pdf]

microRNA-210-3p depletion by CRISPR/Cas9 promoted tumorigenesis through revival of TWIST1 in renal cell carcinoma

SUPPLEMENTARY FIGURES AND TABLES

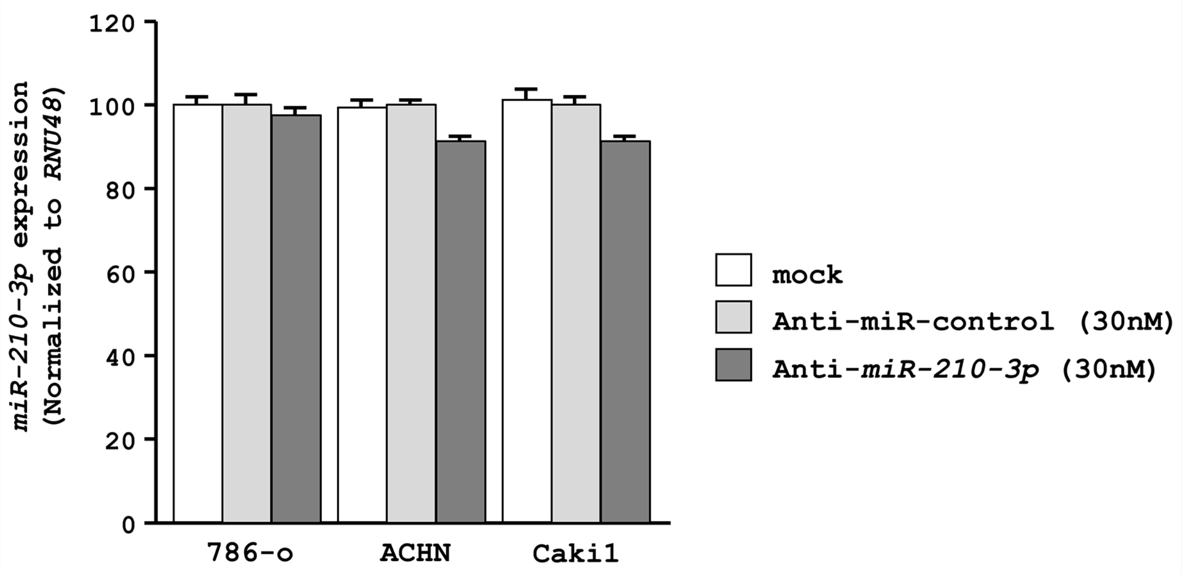

Supplementary Figure 1: The expression levels of *miR-210-3p* in RCC cell lines transfected with anti-miRNA. qRT-PCR data showing that the expression levels of *miR-210-3p* were not decreased by anti-miRNA in 786-o, A498, and Caki2 RCC cell lines.

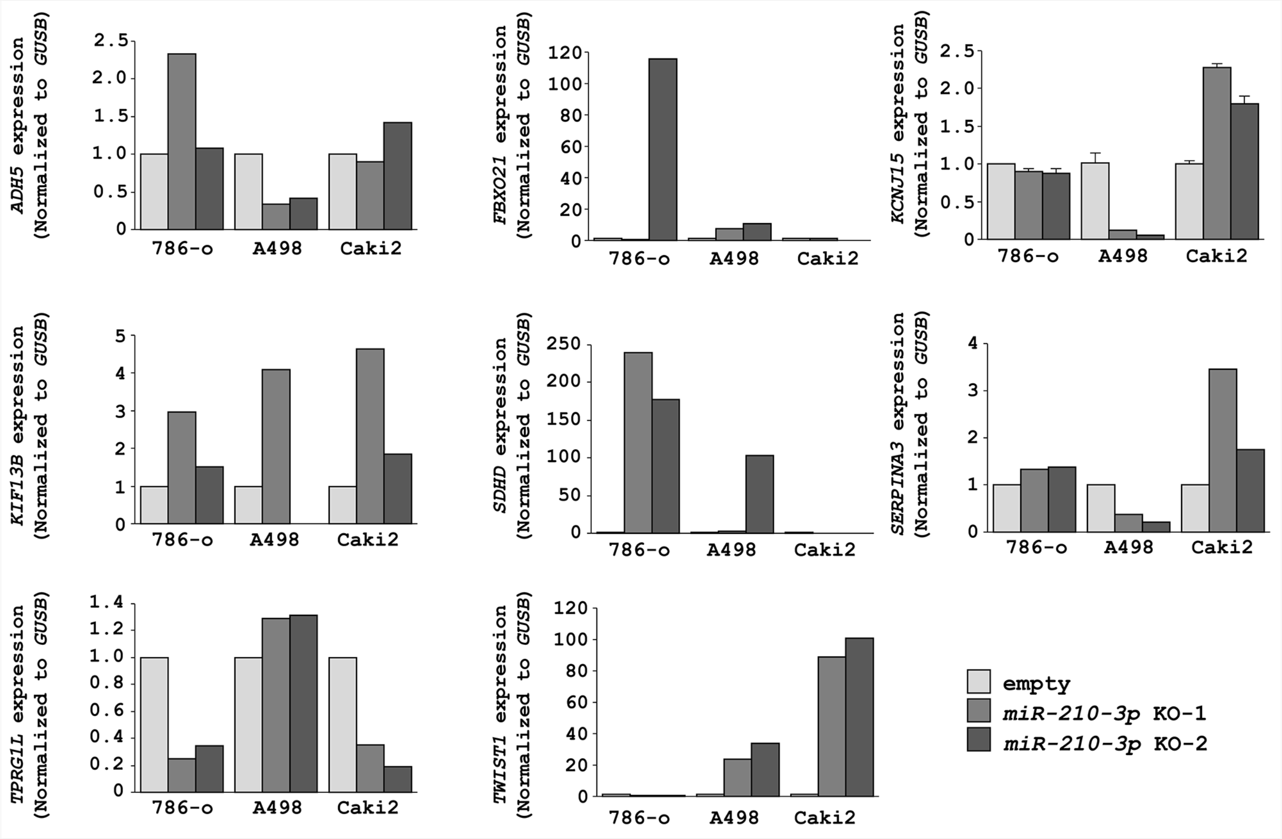

Supplementary Figure 2: mRNA expression levels of the candidate target genes of *miR-210-3p*. qRT-PCR showing the expression levels of *ADH5*, *FBXO21*, *KCNJ15*, *KIF13B*, *SDHD*, *SERPINA3*, *TPRG1L*, *TWIST1* in *miR-210-3p*-depleted 786-o, A498 and Caki2 cell lines.

Supplementary Table 1: Patient characteristics

**Renal carncer**		
Total number	40	
Median Age (range)	66.5	(41-89)
Gender		
Male	28	70.0%
Female	12	30.0%
Pathological tumor stage		
pT1a	20	50.0%
pT1b	13	32.5%
pT2	0	0%
pT3a	4	10.0%
pT3b	3	7.5%
pT4	0	0%
Tumor grade		
G1	3	7.5%
G2	30	75.0%
G3	6	15.0%
unknown	1	2.5%
Metastasis		
M 0	36	90.0%
M 1	2	5.0%
unknown	2	5.0%
Venous invasion		
v 0	25	62.5%
v 1	15	37.5%

Supplementary Table 2: Sequences of each gene

Gene name	NCBI GeneID	Species	Forward	Reverse
GUSB	2990	Human	CGTCCCACCTAGAATCTGCT	TTGCTCACAAAGGTCACAGG
KCNJ15	3772	Human	CCGCGTCATGTCCAAGAGT	TGCAGGTAGAGTAGGTATATGCC
TWIST	7291	Human	GTCCGCAGTCTTACGAGGAG	GCTTGAGGGTCTGAATCTTGCT
SERPINA3	12	Human	CCTGAAGGCCCCTGATAAGAA	GCTGGACTGATTGAGGGTGC
FBXO21	23014	Human	GAGGTGGCCTTCCCTTATGAA	CCCAGCTTTTTGCCGAACTTTA
KIF13B	23303	Human	AGCCGAAGGTGTTTGCTTATG	TGAGTTCGTTCAAAGAGTCCACT
SDHD	6392	Human	ATTTCTTCAGGACCGACCTATCC	CAGCCTTGGAGCCAGAATG
TPRG1L	127262	Human	CAACGCAGTAGACACCATTTCC	CCCAAAACCTTCTCGCTTGTT
ADH5	128	Human	ATGGCGAACGAGGTTATCAAG	CATGTCCCAAGATCACTGGAAAA
RAD52	5893	Human	CCAGAAGGTGTGCTACATTGAG	ACAGACTCCCACGTAGAACTTG
EFNA3	1944	Human	CATGCGGTGTACTGGAACAG	AGATAGTCGTTCACGTTCACCT
